# Anchoring CoFe_2_O_4_ Nanoparticles on N‐Doped Carbon Nanofibers for High‐Performance Oxygen Evolution Reaction

**DOI:** 10.1002/advs.201700226

**Published:** 2017-08-07

**Authors:** Tongfei Li, Yinjie Lv, Jiahui Su, Yi Wang, Qian Yang, Yiwei Zhang, Jiancheng Zhou, Lin Xu, Dongmei Sun, Yawen Tang

**Affiliations:** ^1^ Jiangsu Key Laboratory of New Power Batteries Jiangsu Collaborative Innovation Centre of Biomedical Functional Materials School of Chemistry and Materials Science Nanjing Normal University Nanjing 210023 China; ^2^ Jiangsu Optoelectronic Functional Materials and Engineering Laboratory School of Chemistry and Chemical Engineering Southeast University Nanjing 211189 China; ^3^ School of Chemistry and Chemical Engineering Southeast University Nanjing 211189 China

**Keywords:** carbon nanofibers, CoFe_2_O_4_ nanoparticles, electrospinning, oxygen evolution reaction

## Abstract

The exploration of earth‐abundant and high‐efficiency electrocatalysts for the oxygen evolution reaction (OER) is of great significant for sustainable energy conversion and storage applications. Although spinel‐type binary transition metal oxides (AB_2_O_4_, A, B = metal) represent a class of promising candidates for water oxidation catalysis, their intrinsically inferior electrical conductivity exert remarkably negative impacts on their electrochemical performances. Herein, we demonstrates a feasible electrospinning approach to concurrently synthesize CoFe_2_O_4_ nanoparticles homogeneously embedded in 1D N‐doped carbon nanofibers (denoted as CoFe_2_O_4_@N‐CNFs). By integrating the catalytically active CoFe_2_O_4_ nanoparticles with the N‐doped carbon nanofibers, the as‐synthesized CoFe_2_O_4_@N‐CNF nanohybrid manifests superior OER performance with a low overpotential, a large current density, a small Tafel slope, and long‐term durability in alkaline solution, outperforming the single component counterparts (pure CoFe_2_O_4_ and N‐doped carbon nanofibers) and the commercial RuO_2_ catalyst. Impressively, the overpotential of CoFe_2_O_4_@N‐CNFs at the current density of 30.0 mA cm^−2^ negatively shifts 186 mV as compared with the commercial RuO_2_ catalyst and the current density of the CoFe_2_O_4_@N‐CNFs at 1.8 V is almost 3.4 times of that on RuO_2_ benchmark. The present work would open a new avenue for the exploration of cost‐effective and efficient OER electrocatalysts to substitute noble metals for various renewable energy conversion/storage applications.

Electrocatalytic oxygen evolution reaction (OER, 4OH^−^ → 2H_2_O + O_2_ + 4*e* ^−^) has stimulated considerable research interests due to its pivotal roles in various sustainable energy conversion and storage devices, such as regenerative fuel cells, solar cells, rechargeable metal–air batteries, and water electrolysis.^1^, ^2^, ^3^, ^4^ However, the high overpotential and sluggish reaction kinetics of OER dramatically restricts the overall efficiency of energy conversion. Therefore, enormous efforts have been devoted to developing efficient electrocatalysts to reduce the overpotential and expedite the kinetics of the OER.^5^, ^6^, ^7^ To date, commercial electrocatalysts for OER still rely on the precious metal oxides, such as IrO_2_ and RuO_2_. Unfortunately, their extremely high costs and scarce reserve as well as insufficient long‐term stability greatly impede their widespread applications and scalable commercialization in electrochemical energy devices.^8^, ^9^, ^10^, ^11^ As such, it is extremely important to exploit earth‐abundant and low‐cost alternative catalysts with high activity and durability comparable or even superior to IrO_2_/RuO_2_ benchmarks for OER.

It is well‐documented that the spinel‐type binary transition metal oxides (AB_2_O_4_, A, B = metal) represent a class of promising candidates for water oxidation catalysis because of their high abundance, low toxicity, rich redox chemistry, and superior stability.^12^, ^13^, ^14^, ^15^, ^16^, ^17^ However, their intrinsically inferior electrical conductivity during electrocatalysis process exerts remarkably negative impacts on their electrochemical performances. To address these issues, one of the effective strategies is to hybridize the AB_2_O_4_ nanocatalysts with conductive carbon‐based substrates (i.e., activated carbon, carbon nanotubes/nanofibers, and graphene) in order to improve their conductivity and electrochemical stability, as well as facilitate charge transfer of the integrated system, thus giving rise to an enhanced OER performance.^18^, ^19^, ^20^, ^21^, ^22^, ^23^, ^24^ Moreover, heteroatom‐doping, such as N‐doping, into nanocarbon could effectively improve the electronic conductivity and modulate the electronic structures of the carbon matrix, which is beneficial to boost the OER activity.^25^, ^26^ Among various carbon‐based supports, 1D carbon nanofibers have been attracting enormous attention in electrochemical energy‐related fields due to their large exposed surfaces, shortened distance for mass diffusion and direct efficient pathway for electron transport.^27^, ^28^, ^29^ Therefore, it is reasonably anticipated that the integration of catalytically active AB_2_O_4_ nanostructures with highly conductive carbon nanofibers into a nanohybrid could achieve a satisfactory OER performance with high activity and structural robustness. However, for the immobilization of nanocatalysts on carbon supports, previous protocols generally involved multiple complicated synthetic procedures and the nanocatalysts may suffer from aggregation or detachment from the support.^30^ To this end, it is highly urgent to explore simple and economical routes to strongly couple AB_2_O_4_ nanostructures with carbon nanofibers, yet still remains challenging. Fortunately, electrospinning represents a feasible and effective synthetic technique to fabricate metal oxide/carbon‐based nanofibers with large surface area, small and uniform grain size, and high morphological uniformity. Moreover, the electrospinning technique is more appealing and promising for practical applications due to its ease of operation, environmentally benign, and large scale production capability.^31^, ^32^, ^33^

Herein, we demonstrate a facile and reliable electrospinning strategy to synthesize CoFe_2_O_4_ nanoparticle‐embedded into N‐doped carbon nanofibers (denoted as CoFe_2_O_4_@N‐CNFs) with high yield and uniformity, which is schematically illustrated in **Figure** **1**. Briefly, the precursor solution containing polyvinylpyrrolidone (PVP), *N*,*N*‐Dimethylformamide (DMF), Co(NO_3_)_2_, and Fe(NO_3_)_3_ was initially electrospun into a nanofiber membrane. Subsequently, the as‐spun uniform polymer nanofibers were stabilized at 250 °C for 3 h in air atmosphere followed by calcination at 600 °C for 3 h in N_2_ atmosphere. During the calcination process, the PVP nanofibers would be carbonized into N‐doped carbon nanofibers and Co/Fe nitrates would be transformed into spinel‐phased CoFe_2_O_4_ nanoparticles. Benefitting from the 1D structural feature and synergy of CoFe_2_O_4_ species of and N‐doped carbon nanofibers, the as‐synthesized CoFe_2_O_4_@N‐CNFs exhibits remarkable OER performance in 0.1 m KOH medium with relatively low overpotential, much improved current density, favorable reaction kinetics, and outstanding long‐term stability, as compared with the single‐component counterparts (pure CoFe_2_O_4_ and N‐CNFs) and the commercial RuO_2_ electrocatalyst.

**Figure 1:**
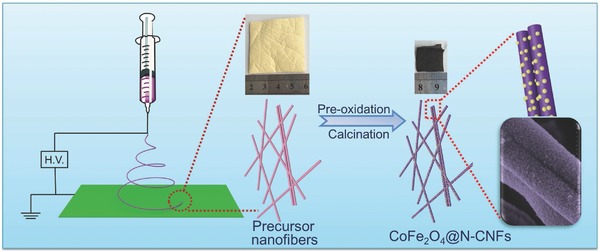
Schematic illustration of the overall synthesis of the CoFe_2_O_4_@N‐CNFs.

Figure S1 (Supporting Information) presents the typical scanning electron microscopy (SEM) images of the as‐prepared polymer nanofibers. It is clearly observed that the resultant polymer nanofibers with smooth surface and uniform diameters are randomly oriented and highly interconnected, forming 3D continuous networks with mechanical robustness. Such intriguing structural feature is favorable to electron transfer and mass diffusion. Higher magnification SEM images indicate that the average diameter of the polymer nanofibers is around 1.0 µm and the length is up to tens of micrometers. As shown in **Figure** **2**a,b the fibers could preserve the original 1D fibrous structure and the interwoven network structure could be still well maintained after the subsequent two‐step annealing processes, whereas the average diameter of the obtained CoFe_2_O_4_@N‐CNFs is reduced to ≈250 nm, approximately a quarter of that of the parent nanofibers due to the thermal decomposition of PVP matrix and pyrolysis of metal–salt precursors. Closer observations (Figure 2c,d) demonstrate that the surface of the CoFe_2_O_4_@N‐CNFs becomes obviously rough and numerous CoFe_2_O_4_ nanoparticles are homogeneously dispersed in the nanofiber supports without any agglomeration.

**Figure 2:**
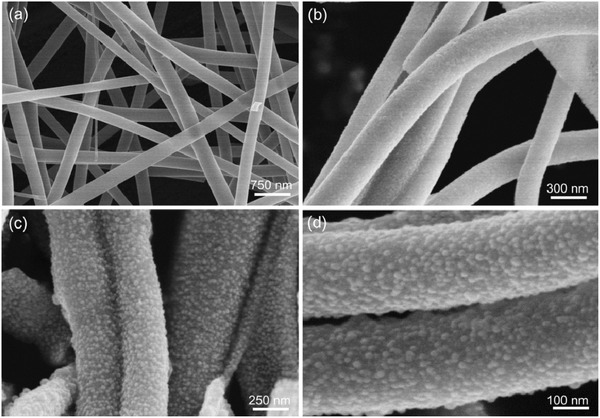
a–d) Representative SEM images of the obtained CoFe_2_O_4_@N‐CNFs with different magnifications.

In consistent with the aforementioned SEM results, representative transmission electron microscopy (TEM) images (**Figure** **3**a,b) verify that CoFe_2_O_4_ nanoparticles are uniformly dispersed in the fibrous carbon matrix. Particle size statistics (inset of Figure 3b) of the CoFe_2_O_4_ grains reveals a narrow size distribution and an average size of around 31.4 nm. A high‐resolution TEM image (HRTEM) shown in Figure 3c further confirms that CoFe_2_O_4_ nanoparticles are discretely dispersed within the carbon scaffolds, without obvious aggregation. Selected area electron diffraction (SAED) pattern (inset of Figure 3c) of an individual CoFe_2_O_4_ nanoparticle demonstrates the polycrystalline feature of the CoFe_2_O_4_ nanoparticles. The lattice fringe (Figure 3d) recorded from square area marked in Figure 3c is clearly measured to be 0.21 nm, corresponding to interplanar distance of the (400) plane of spinel‐phased CoFe_2_O_4_. The high‐angle annular dark‐field scanning TEM (HAADF‐STEM) image and elemental mapping images (Figure 3e) suggest the homogeneous distribution of Co, Fe, O, and C throughout the fibrous nanocomposite.

**Figure 3:**
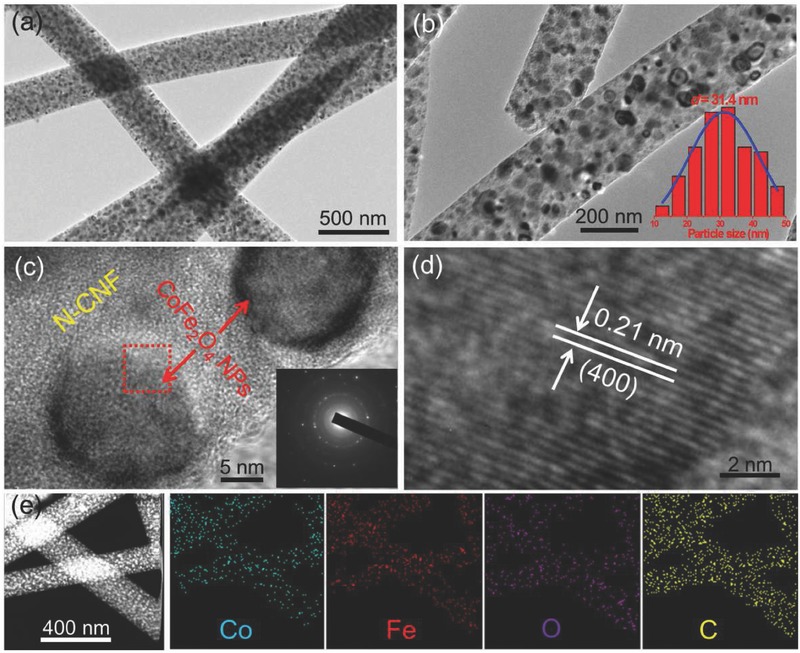
a,b) TEM images, c,d) HRTEM images, e) HAADF‐STEM image, and elemental mapping images of the CoFe_2_O_4_@N‐CNFs. Insets of (b) and (c) show the particle size distribution and SAED pattern of the CoFe_2_O_4_ nanoparticles, respectively.

X‐ray diffraction (XRD) pattern of the CoFe_2_O_4_@N‐CNFs is presented in **Figure** **4**a. All of the diffraction peaks can be attributed to the spinel‐type CoFe_2_O_4_, whose unit cell structure is constructed by CoO_4_ tetrahedra and FeO_6_ octahedra (Figure 4b). The sharp diffraction peaks imply the high crystallinity of the CoFe_2_O_4_. The energy dispersive X‐ray spectroscopy (EDS) shown in Figure 4c suggests the presence of Co, Fe, O, and C in the obtained CoFe_2_O_4_@N‐CNFs with the Fe/Co molar ratio of ≈2.04, which is in agreement with the stoichiometric ratio of 2. It is noteworthy that the Cu signal comes from the copper grid. The carbon content in the CoFe_2_O_4_@N‐CNFs acquired from the thermogravimetric analysis (TGA) in Figure 4d is quantitatively to be 4.7 wt%. The degree of graphitization of the carbon nanofibers is investigated by Raman spectrum, as illustrated in Figure 4e. Two well‐defined peaks can be observed at 1358 and 1588 cm^−1^, which are assigned to the D and G bands of carbon materials, respectively. It is well‐established that the D band arises from the disordered or defect carbon and the G band originates from the sp^2^‐hybridized graphitic carbon. The intensity ratio between D band and G band (*I*_D_/*I*_G_) generally reflects the graphitization degree of carbon materials and a lower value to *I*_D_/*I*_G_ indicates a higher graphitization degree.^34^, ^35^, ^36^, ^37^, ^38^ Here the *I*_D_/*I*_G_ ratio of CoFe_2_O_4_@N‐CNFs is calculated to be 0.83, suggesting a well‐crystallized graphitic carbon in the carbon nanofibers. Such a high graphitization degree is beneficial to improve the electronic conductivity of the hybrid nanofibers. The N_2_ adsorption–desorption isotherms (Figure 4f) of the CoFe_2_O_4_@N‐CNFs can be categorized as type‐IV isotherms with a noticeable hysteresis loop, indicating the presence of mesopores (2–50 nm). The Brunauer–Emmett–Teller (BET) surface area is measured to be 52.9 m^2^ g^−1^.

**Figure 4:**
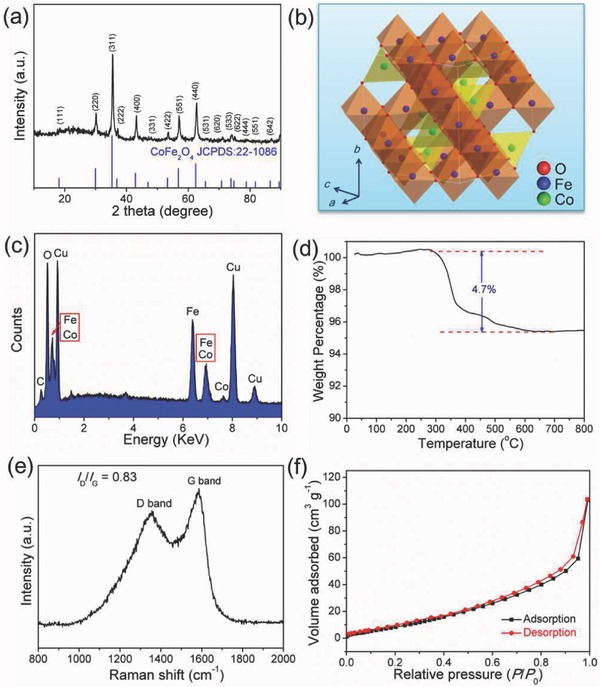
Compositional characterization of the as‐prepared CoFe_2_O_4_@N‐CNFs. a) XRD pattern, b) crystal structure of spinel‐phased CoFe_2_O_4_, c) EDS, d) TGA curve, e) Raman spectrum, and f) N_2_ adsorption–desorption isotherms.

The chemical compositions and valence states of the constituent elements in the as‐fabricated CoFe_2_O_4_@N‐CNFs are investigated through X‐ray photoelectron spectroscopy (XPS) technique. As displayed in **Figure** **5**a, the survey‐scan spectrum manifests that the sample is composed of Co, Fe, C, N, and O elements. The existence of N can be ascribed to the pyrolysis of PVP during the calcination process.^39^, ^40^ The high‐resolution Fe 2p spectrum (Figure 5b) exhibits two characteristic peaks at 711.0 eV (Fe 2p_3/2_) and 724.5 eV (Fe 2p_1/2_) as well as a minor peak at 718.5 eV (shake‐up satellite peak of Fe 2p_3/2_), indicating the oxidation state of Fe^3+^. The high‐resolution Co 2p spectrum (Figure 5c) can be deconvoluted into four peaks, which are corresponding to Co 2p_3/2_ (779.6 eV), Co 2p_3/2_ satellite peak (785.8 eV), Co 2p_1/2_ (795.1 eV), and Co 2p_1/2_ satellite peak (801.6 eV), respectively. The presence of Co 2p_3/2_ and Co 2p_1/2_ main peaks and their shake‐up satellite peaks suggest the oxidation state of Co^2+^. The high‐resolution C 1s spectrum (Figure 5d) displays a prominent nonoxygenated C—C peak (284.6 eV) and a weak C—O peak (286.2 eV). The C—O peak may be arisen from the covalent coupling between the CoFe_2_O_4_ and C support or from some oxygen‐containing groups on the surface of the carbon nanofibers. The high‐resolution N 1s spectrum (Figure 5e) can be well fitted into four peaks and assigned to the pyridinic N (398.6 eV), pyrrolic N (399.9 eV), graphitic N (400.9 eV), and oxidized N (401.9 eV), respectively. Figure 5f shows the schematic representation of the four types of nitrogen configurations in carbon matrix. The incorporation of N into carbon nanofibers can not only effectively enhance the overall electrical conductivity of carbon nanofibers but also generate some defects or vacancies among carbon nanofibers, therefore, affording numerous active sites for electrocatalysis and thus expediting the reaction kinetics.^41^, ^42^, ^43^, ^44^

**Figure 5:**
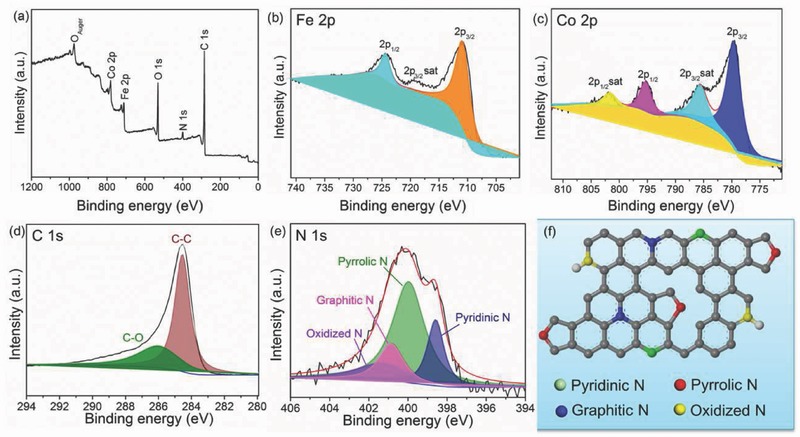
XPS spectra of the CoFe_2_O_4_@N‐CNFs. a) Survey scan spectrum, b) Fe 2p region, c) Co 2p region, d) C 1s, e) N 1s, and f) schematic configurations of N with different chemical states in C matrix.

Inspired by the CoFe_2_O_4_ nanoparticles anchored on highly conductive carbon nanofiber networks, the electrocatalytic performance of the as‐synthesized CoFe_2_O_4_@N‐CNF hybrid toward OER was appraised in 0.1 m KOH solution using a standard three‐electrode system. For comparison, pure CoFe_2_O_4_ (Figure S2, Supporting Information), N‐doped carbon nanofibers (N‐CNFs, Figure S3, Supporting Information), and commercial RuO_2_ were also evaluated under the identical measurement conditions. The textural properties of the CoFe_2_O_4_ and N‐doped carbon nanofibers, including N_2_ adsorption–desorption isotherms and pore‐size distribution curves, are presented in Figure S4 of the Supporting Information. **Figure** **6**a shows the typical *IR*‐corrected linear sweep voltammetry (LSV) curves of the four catalysts obtained at a scan rate of 5 mV s^−1^ and 1600 rpm rotation rate. It can be clearly seen that the LSV curves of pure CoFe_2_O_4_ and N‐CNFs show inconspicuous current densities within the tested potential range, suggesting their negligible activities to OER. In striking contrast, the CoFe_2_O_4_@N‐CNF nanohybrid shows a comparable onset potential with the commercial RuO_2_ catalyst, highlighting the synergistic effect between CoFe_2_O_4_ and N‐CNFs. As we know, the overpotential (η) required to afford a current density of 10.0 mA cm^−2^, approximately the current density for a 10% efficient solar‐to‐fuel conversion device, is an important figures‐of‐merit to evaluate an OER catalyst.^45^, ^46^ The η of the CoFe_2_O_4_@N‐CNFs to achieve a current density of 10.0 mA cm^−2^ is 349 mV, which is almost identical with that of the commercial RuO_2_ catalyst (342 mV). Surprisingly, under the higher current densities, the required overpotentials of the CoFe_2_O_4_@N‐CNFs are significantly lower than those of the commercial RuO_2_ catalyst, as shown in Figure 6b. Specifically, the overpotential of CoFe_2_O_4_@N‐CNFs at the current density of 30.0 mA cm^−2^ is determined to be 408 mV, which negatively shifts 186 mV as compared with the commercial RuO_2_ catalyst. Meanwhile, the CoFe_2_O_4_@N‐CNFs can deliver a much higher current density under the same applied potential, as illustrate in Figure 6c. The CoFe_2_O_4_@N‐CNFs can attain a current density of 56.68 mA cm^−2^ at 1.7 V, which is 2.6 times higher than that of RuO_2_ reference. Similarly, the current density of the CoFe_2_O_4_@N‐CNFs reaches a value of 97.50 mA cm^−2^ at 1.8 V, almost 3.4 times of that on RuO_2_ benchmark. By using cyclic voltammetry (CV) measurement, the electrochemically active surface areas (ECSAs) of the prepared CoFe_2_O_4_@N‐CNFs and commercial RuO_2_ catalyst are evaluated by their electrochemical double‐layer capacitances (*C*_dl_) due to the fact that *C*_dl_ is proportional to the ECSA. Figure S5a,b (Supporting Information) presents the CV curves of the two catalysts recorded in a non‐Faradic potential range under different scan rates. As displayed in Figure S5c (Supporting Information), the *C*_dl_ value of the CoFe_2_O_4_@N‐CNFs is determined to be 20.6 mF cm^−2^, which is 1.4 times higher than that of the commercial RuO_2_ catalyst. This result demonstrates that the synthesized CoFe_2_O_4_@N‐CNFs could afford a larger number of catalytically active sites and thus an improved OER activity.

**Figure 6:**
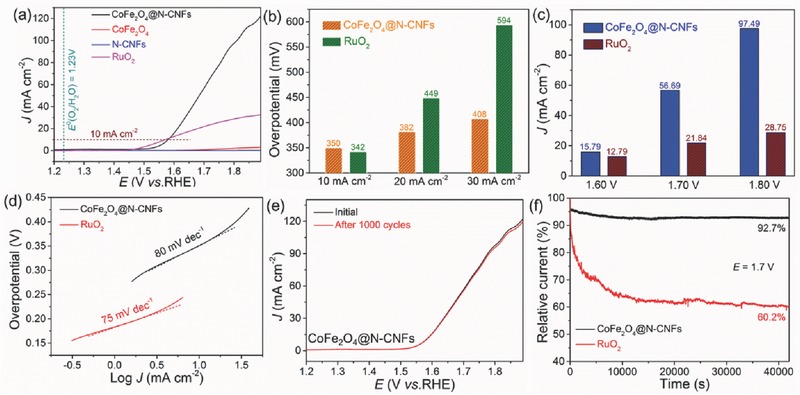
Comparison of OER performances of different catalysts. a) LSV polarization curves, b) required overpotentials derived from OER polarization curves at different current densities, c) current densities achieved at different potentials, d) Tafel plots, e) LSV polarization curves of the CoFe_2_O_4_@N‐CNFs before and after 1000 cycles, and f) chronopotentiometry curves of the CoFe_2_O_4_@N‐CNFs and commercial RuO_2_ catalyst.

The electrocatalytic kinetics for OER of the CoFe_2_O_4_@N‐CNFs and the commercial RuO_2_ catalysts are further investigated by Tafel plots, as displayed in Figure 6d. The Tafel slope of the CoFe_2_O_4_@N‐CNFs is identified as 80 mV dec^−1^, which is comparable to that of the RuO_2_ catalyst (75 mV dec^−1^). Moreover, the similar Tafel slope of the two catalysts indicates that both catalysts undergo the same rate‐determining step and reaction pathway toward the OER.^47^, ^48^ As compared with the previously reported nonprecious metal‐based OER electrocatalysts, our CoFe_2_O_4_@N‐CNFs show comparable and even better electrocatalytic properties toward OER in basic solution with relatively low onset potential and Tafel slope, as summarized in Table S1 of the Supporting Information.

The long‐term stability of an electrocatalyst is also a critical parameter for practical applications. As illustrated in Figure 6e, the LSV curve of the CoFe_2_O_4_@N‐CNFs for the OER shows negligible degradation after continuous 1000 CV cycles, indicating its superior operational stability under alkaline test condition. Consistently, the chronopotentiometric curves performed at 1.7 V (Figure 6f) indicate that the current attenuation of the CoFe_2_O_4_@N‐CNFs after 40 000 s is merely 7.3%, whereas the RuO_2_ suffers great activity deterioration, with a current loss of 39.8% after 40 000 s. Furthermore, as evidenced TEM images shown in Figure S6a,b (Supporting Information), the 1D fiber‐like structure of the CoFe_2_O_4_@N‐CNFs after the long‐term stability test could be well retained and CoFe_2_O_4_ nanoparticles are still well dispersed. Particle size statistics (inset of Figure S6b, Supporting Information) further indicates that the average size of the CoFe_2_O_4_ nanoparticles is still centered at ≈32 nm, without obvious aggregation and expansion, thanks to the immobilization effect of carbon nanofiber scaffold. A high‐resolution TEM image (Figure S6c, Supporting Information) further confirms that CoFe_2_O_4_ nanoparticles are firmly dispersed within the carbon scaffolds. The well‐resolved lattice fringe (Figure S6d, Supporting Information) recorded from square area marked in Figure S6c (Supporting Information) is clearly measured to be 0.299 nm, corresponding to interplanar distance of the (220) plane of spinel‐phased CoFe_2_O_4_. The HAADF‐STEM image and elemental mapping images (Figure S6e, Supporting Information) imply the uniform distribution of C, O, Fe, and Co throughout the CoFe_2_O_4_@N‐CNFs after the long‐term stability. All these results unambiguously affirm the structural and chemical stability of the obtained CoFe_2_O_4_@N‐CNFs after long‐term stability test. Taken together, all above results strongly demonstrate that the synthesized CoFe_2_O_4_@N‐CNFs possess superior OER performance with relatively low overpotential, enhanced activity, satisfied kinetics, and better stability, endowing it a promising efficient OER electrocatalyst for future applications.

The outstanding OER performance of the prepared CoFe_2_O_4_@N‐CNFs can be mainly attributed to the unique structural feature and the synergistic effect between the well‐dispersed tiny CoFe_2_O_4_ nanoparticles and the nitrogen‐doped graphitic carbon nanofibers, as illustrated in **Figure** **7**. To be specific, (1) the numerous homogeneously distributed CoFe_2_O_4_ nanoparticles could afford a high density of OER active sites on the surface; (2) the hybridization of CoFe_2_O_4_ nanoparticles with nitrogen‐doped graphitic carbon could not only remarkably endow the composite with good conductivity for charge transfer during electrochemical process, but also firmly immobilize the CoFe_2_O_4_ nanoparticles, preventing their detachment or aggregation; (3) the nitrogen doping in carbon nanofibers could provide more catalytically active sites for OER; (4) the network constructed by interconnected 1D nanofibers could offer continuous 3D pathways for mass diffusion and electron transport. Furthermore, the strong coupling between CoFe_2_O_4_ nanoparticles and nitrogen‐doped carbon nanofibers may give rise to a synergistic effect and thus an improved OER activity. By integrating all above advantages, the as‐prepared CoFe_2_O_4_@N‐CNFs exhibit impressive OER performance with exceptional activity and excellent stability.

**Figure 7:**
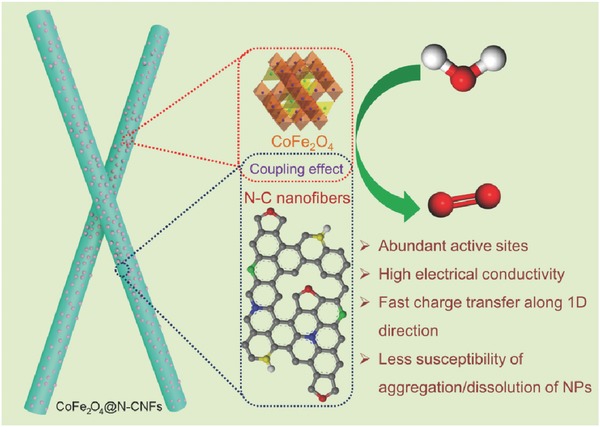
A schematic illustration of the structural and compositional advantages of the synthesized CoFe_2_O_4_@N‐CNFs as an efficient OER electrocatalyst.

In conclusion, we have presented a simple and scalable electrospinning strategy for the concurrent synthesis of CoFe_2_O_4_ nanoparticles homogeneously embedded in N‐doped carbon nanofibers. Compared with the single component counterparts (pure CoFe_2_O_4_ and N‐doped carbon nanofibers) and commercial RuO_2_ catalyst, the synthesized CoFe_2_O_4_@N‐CNFs are demonstrated to be an efficient earth‐abundant OER electrocatalyst with a low overpotential, a large current density, a small Tafel slope, and long‐term durability in alkaline solution. The improved catalytic performances are believed to originate from the unique 1D structural feature and the synergy between the constituent components. Considering the cost‐effectiveness, facile, and reliable fabrication process, and outstanding catalytic performance, the CoFe_2_O_4_@N‐CNFs may hold great potential to in future energy conversion and storage devices. More importantly, the present versatile synthetic strategy may stimulate the rational design of other metal oxides/carbon nanofibers through the similar one‐step concurrent growth method for diverse applications in the future.

## Experimental Section

*Synthesis of CoFe_2_O_4_@N‐CNFs*: For the typical electrospinning synthesis of CoFe_2_O_4_@N‐CNFs, 1.0 g PVP (average *M*_w_ = 1 300 000, Alfa Aesar) was initially dissolved in 10 mL DMF (Sinpharm Chemical Reagent) with vigorous stirring for 6 h to obtain a homogeneous solution. Subsequently, 1 mmol Co(NO_3_)_2_·6H_2_O and 2 mmol Fe(NO_3_)_3_·9H_2_O were introduced into the above solution with rapid stirring for another 12 h. The resultant red‐brown viscous liquid was loaded into a plastic syringe equipped with a 20‐gauge needle that was electrically connected to a high voltage power supply. During the electrospinning process, the flow rate of solution was set at 1.0 mL h^−1^ controlled by a syringe pump. A high voltage of 18 KV was applied between the needle and the fiber collector, namely, aluminum foil. The distance between the needle tip and the aluminum foil was 18 cm. The as‐spun fiber membrane was first stabilized in air at 250 °C for 3 h with the heating rate of 1 °C min^−1^. Then the temperature was increased to 600 °C with a heating rate of 5 °C min^−1^ and held for 3 h under N_2_ atmosphere to obtain CoFe_2_O_4_@N‐CNFs.

For comparison, pure CoFe_2_O_4_ and N‐doped carbon nanofibers were also synthesized. For the synthesis of pure CoFe_2_O_4_, the as‐obtained CoFe_2_O_4_@N‐CNFs were calcinated at 600 °C in air for 3 h to completely remove the carbon. For the synthesis of N‐CNFs, the synthetic protocol is similar to that for the synthesis of CoFe_2_O_4_@N‐CNFs, without adding Co and Fe sources into the precursor.

*Characterization*: XRD measurements were performed on a Model D/max‐rC X‐ray diffractometer with Cu Kα radiation (λ = 1.5406 Å). TEM images and HRTEM images were acquired on a JEOL JEM‐2100F transmission electron microscopy operated at an accelerating voltage of 200 kV. Energy dispersive spectrum (EDS), HAADF‐STEM, and elemental mapping images were conducted on an FEI Tecnai G2 F20 microscope, which is built as an accessory on the JEOL JEM‐2100F. Field‐emission scanning electron microscopy images were taken on a JEOL JSM7500F. XPS measurements were conducted on a Thermo VG Scientific ESCALAB 250 spectrometer with an Al Kα radiator. TGA was carried out on a NetzschSTA449C thermal analyzer at a heating rate of 10 °C min^−1^ under air. The BET specific surface area was analyzed at 77 K by Micromeritics ASAP 2050 instrument.

*Electrochemical Measurements*: All electrochemical experiments were performed using a conventional three‐electrode system on a CHI 660 electrochemical analyzer. In a three‐electrode system, the catalyst‐modified glassy carbon electrode (3 mm in diameter) was used as a working electrode, a Pt foil and a saturated calomel electrode served as the counter and reference electrode, respectively. The catalyst ink was prepared by ultrasonically dispersing the mixture of 4 mg of catalyst, 0.5 mL anhydrous ethanol, 1.5 mL distilled water, and 5 µL of 5 wt% Nafion solution. To immobilize the catalyst on the working electrode, 20 µL of the catalyst ink was dropped onto the glassy carbon electrode and dried under ambient condition. All electrochemical measurements were performed in N_2_‐saturated 0.1 m KOH solution, LSV tests were conducted at a sweep rate of 5 mV s^−1^ under 30 ± 1 °C.

## Conflict of Interest

The authors declare no conflict of interest.

## Supporting information

Supplementary
