# Supplementary material for: Anchoring CoFe2O4 Nanoparticles on N‐Doped Carbon Nanofibers for High‐Performance Oxygen Evolution Reaction
Source: Adv Sci (Weinh). 2017 Aug 7;4(11):1700226. doi: 10.1002/advs.201700226 (PMC5700636; doi:10.1002/advs.201700226)
Supplement: Supplementary file 1 — Supplementary [file ADVS-4-na-s001.pdf]

Copyright WILEY-VCH Verlag GmbH & Co. KGaA, 69469 Weinheim, Germany, 2017.

ADVANCED SCIENCE
Open Access

Supporting Information

for *Adv. Sci.*, DOI: 10.1002/advs.201700226

Anchoring $CoFe_2O_4$ Nanoparticles on N-Doped Carbon Nanofibers for High-Performance Oxygen Evolution Reaction

Tongfei Li, Yinjie Lv, Jiahui Su, Yi Wang, Qian Yang, Yiwei Zhang, Jiancheng Zhou, Lin Xu, Dongmei Sun, and Yawen Tang**

Copyright WILEY-VCH Verlag GmbH & Co. KGaA, 69469 Weinheim, Germany, 2016.

Supporting Information

Anchoring $CoFe_2O_4$ nanoparticles on N-doped carbon nanofibers for high-performance oxygen evolution reaction

Tongfei Li, Yinjie Lv, Jiahui Su, Yi Wang, Qian Yang, Yiwei Zhang, Jiancheng Zhou, Lin Xu, Dongmei Sun, and Yawen Tang**

T. Li, Y. Lv, J. Su, Y. Wang, Q. Yang, Prof. L. Xu, Prof. D. Sun, Prof. Y. Tang
Jiangsu Key Laboratory of New Power Batteries, Jiangsu Collaborative Innovation Centre of Biomedical Functional Materials, School of Chemistry and Materials Science, Nanjing Normal University, Nanjing 210023, China
E-mail: njuxulin@gmail.com (L. Xu); tangyawen@njnu.edu.cn (Y. Tang)

Prof. Y. Zhang
Jiangsu Optoelectronic Functional Materials and Engineering Laboratory, School of Chemistry and Chemical Engineering, Southeast University, Nanjing 211189, China

Prof. J. Zhou
School of Chemistry and Chemical Engineering, Southeast University, Nanjing 211189, China

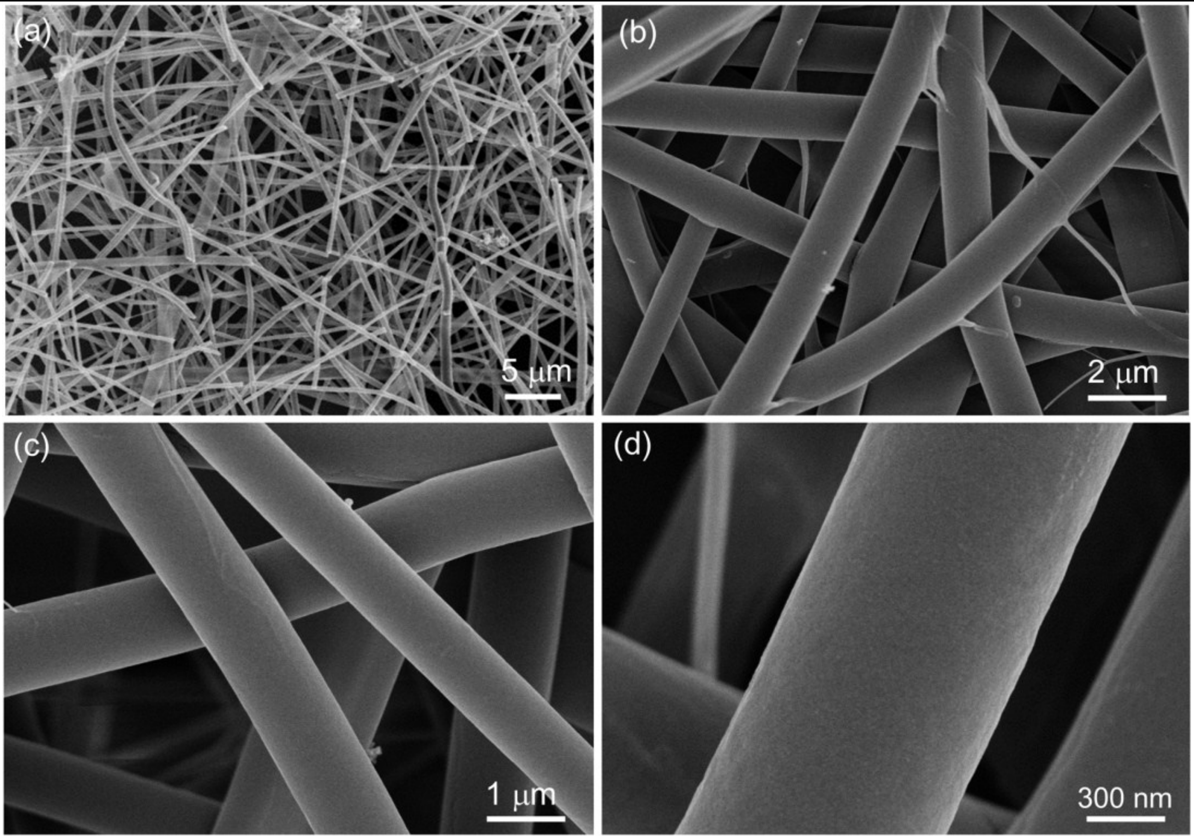

Figure S1. SEM images of the as-spun precursor nanofibers with different magnifications.

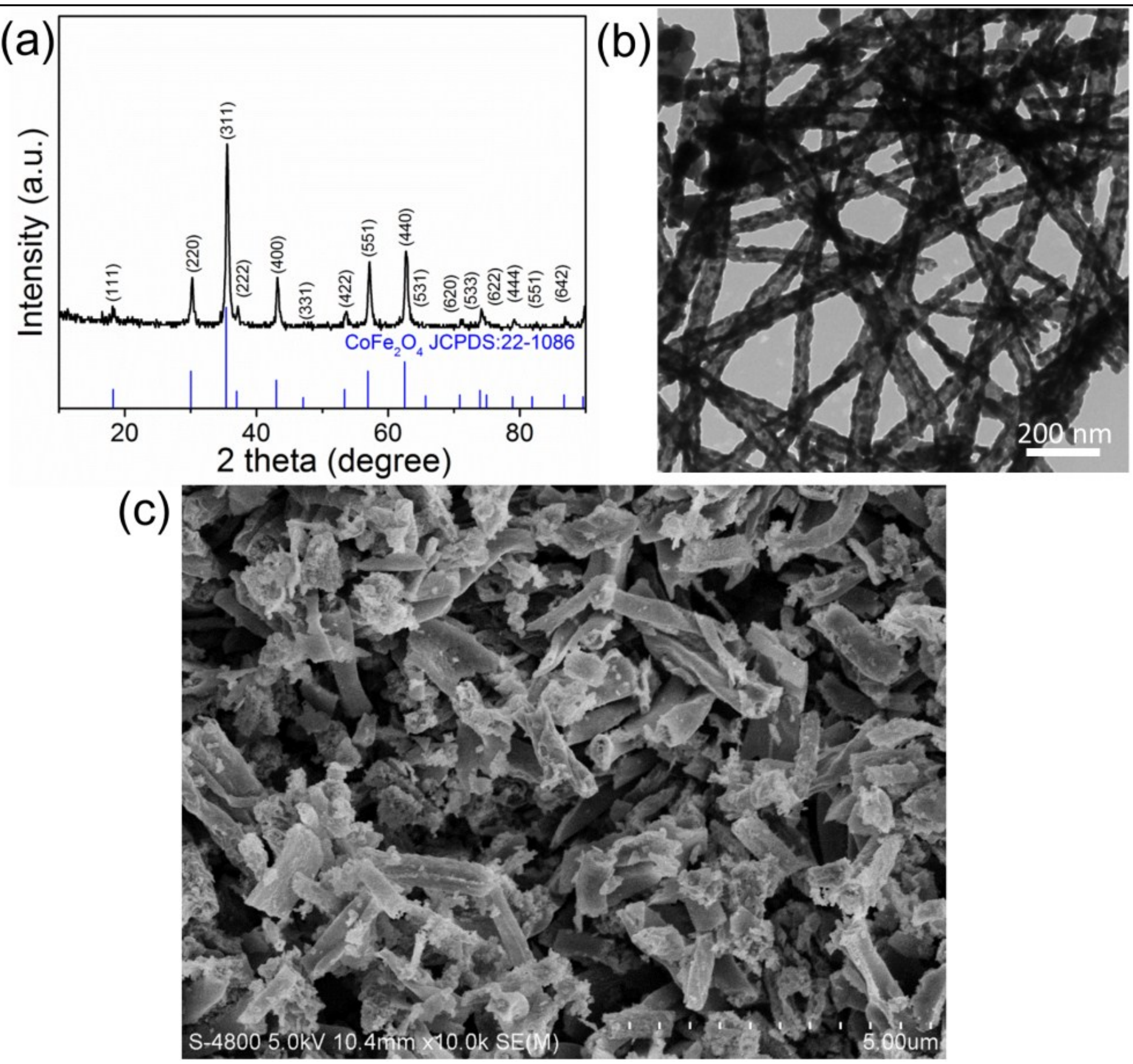

Figure S2. (a) XRD pattern, (b) TEM image and (c) SEM image of the obtained $CoFe_2O_4$ nanostructures obtained through the calcination of preformed $CoFe_2O_4$@N-CNFs at 600 °C for 3 h under air atmosphere.

The XRD pattern indicates its composition of spinel-phased $CoFe_2O_4$. TEM image (Figure S2b) and SEM image (Figure S2c) suggest that the resultant $CoFe_2O_4$ could inherit the structural features well from the parent $CoFe_2O_4$@N-CNFs, *i. e.* fiber-like nanostructure, randomly oriented and highly interconnected.

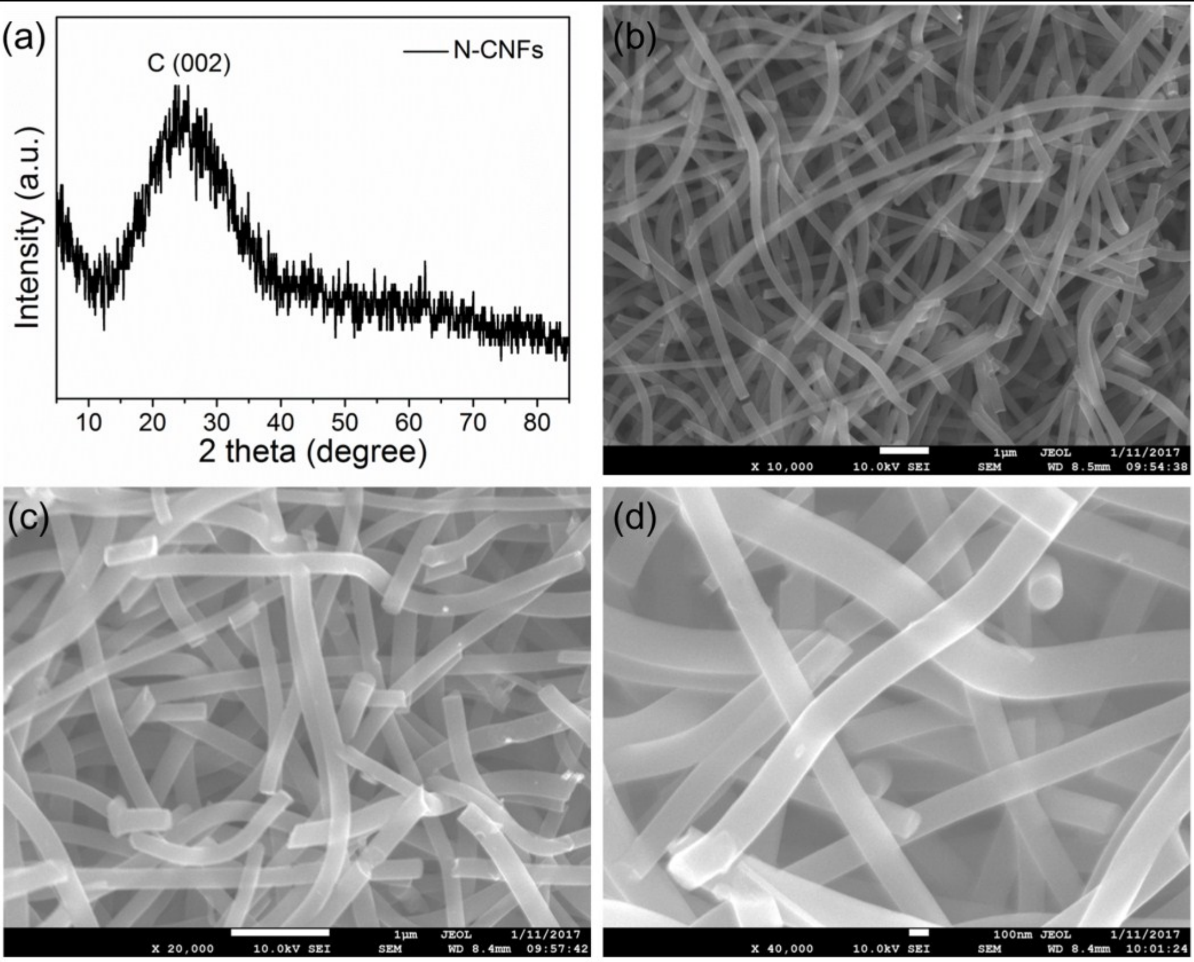

Figure S3. (a) XRD pattern and (b)-(d) typical SEM images of the obtained N-CNFs.

The XRD pattern (Figure S3a) shows the typical diffraction peak at around 25°, which can be indexed to (002) plane of the graphitic carbon. SEM images with different magnifications (Figure S3b-d) demonstrate that the as-spun product is made of 1D nanofibers with smooth surface and uniform diameter of ~ 200 nm.

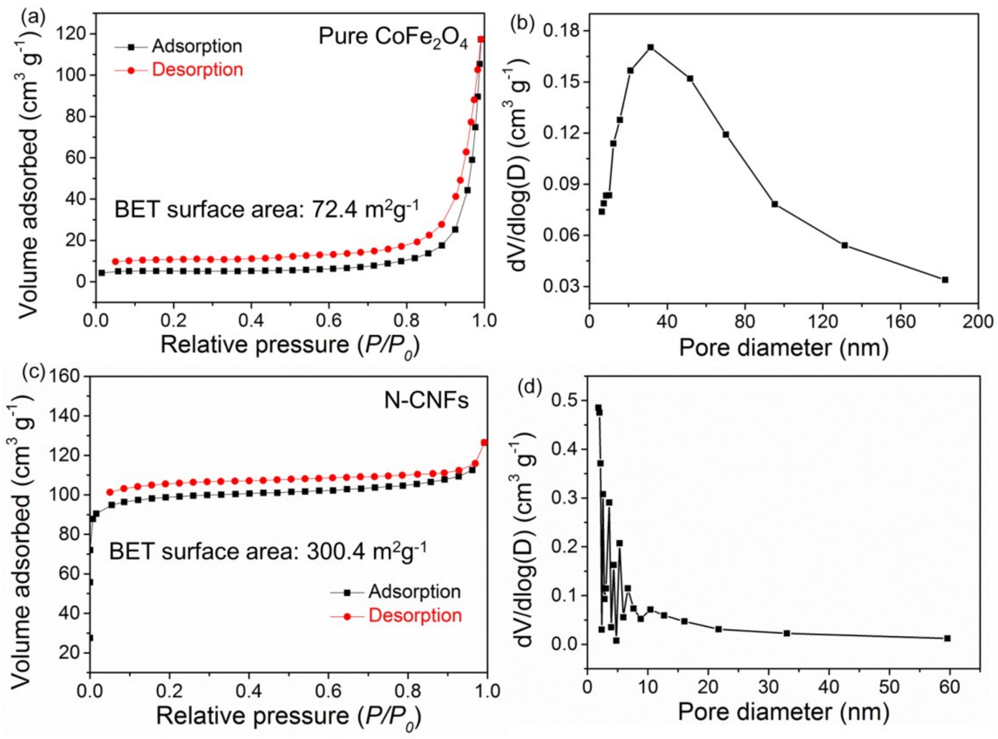

Figure S4. Nitrogen isotherms and corresponding pore-size distribution curves for pure $CoFe_2O_4$ (a-b) and N-CNFs (c-d).

As shown in Figure S4a, the N_2 adsorption-desorption isotherms of $CoFe_2O_4$ can be categorized as type IV, with a typical H4 hysteresis loop nearly parallel over a wide range of p/p_0. Such typical H4 hysteresis loop is generally associated with narrow slit-like pores. The pore size distribution curve (Figure S4b) indicates the predominance of pores ~38 nm in diameter. The BET surface area of the $CoFe_2O_4$ is measured to be 72.4 m^2 g^{-1}. Similarly, the N_2 adsorption-desorption isotherms (Figure S4c) of N-doped carbon nanofibers can be also categorized as type IV, with a typical H4 hysteresis loop nearly parallel over a wide range of p/p_0. It is noteworthy that the N-doped carbon nanofibers exhibit a large adsorption volume at p/p_0. < 0.01, suggesting the existence of micropores (< 2 nm) within the N-doped carbon nanofibers. The generation of micropores may originate from the released gas during the calcination process. The BET surface area of the N-doped carbon nanofibers is measured to be 300.4 m^2 g^{-1}.

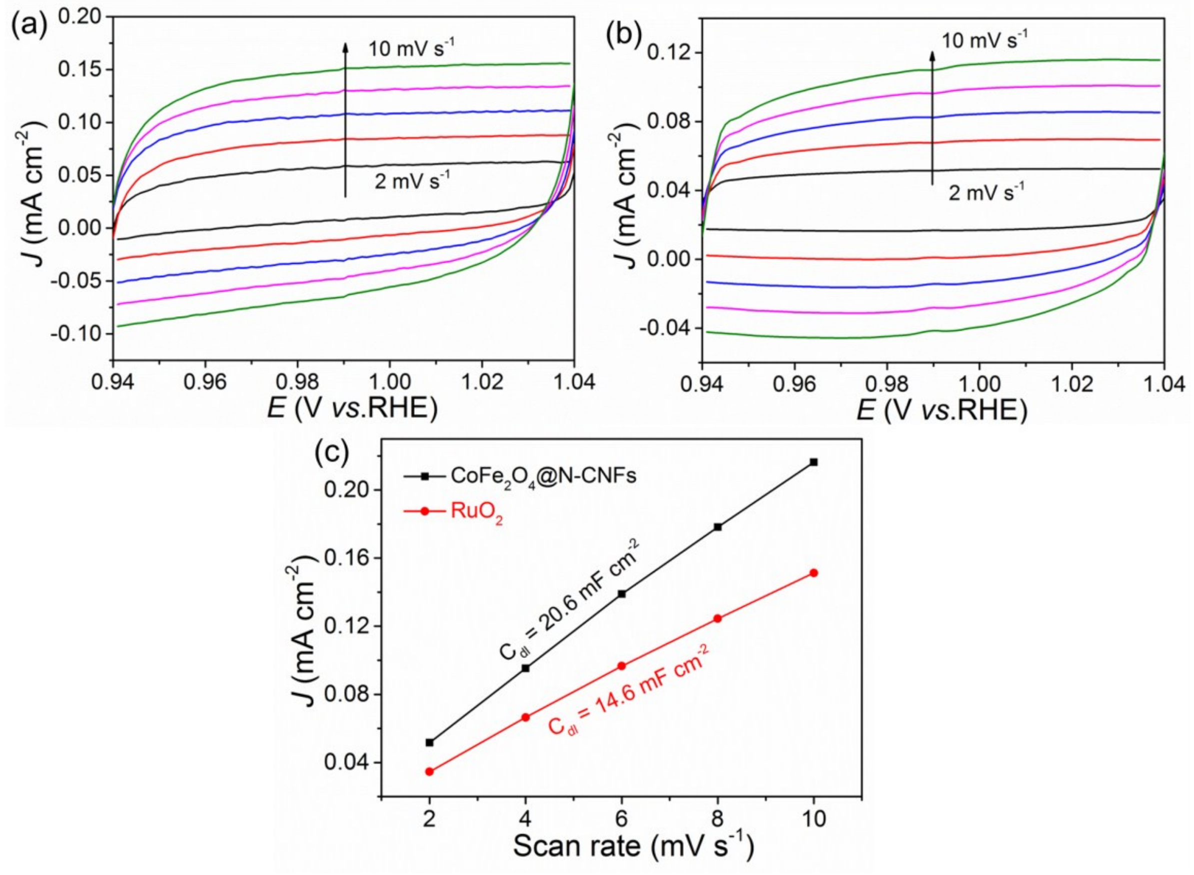

Figure S5. Electrochemical surface area (ESCA) tests of $CoFe_2O_4$@N-CNFs and commercial RuO_2 catalyst recorded in N_2-saturated 0.1 M KOH. (a) CV curves of $CoFe_2O_4$@N-CNFs and (b) commercial RuO_2 catalyst in the non-Faradaic region obtained at different scanning rates. (c) Linear fitting of current density as a function of the scan rate for the different electrodes.

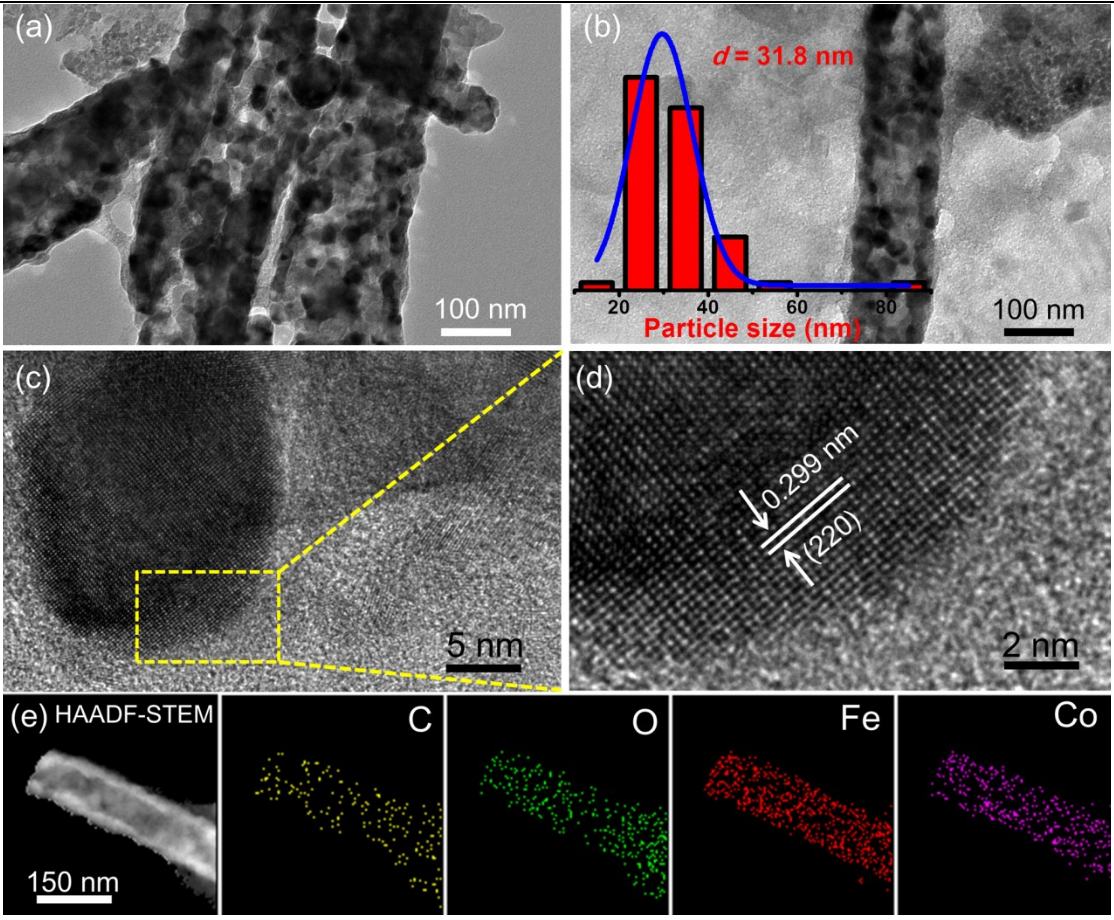

Figure S6. Detailed structural characterization of the $CoFe_2O_4$@N-CNFs after the long-term stability test. (a)-(b) TEM images, (c)-(d) HRTEM images and (e) HAADF-STEM image and elemental mapping images. Inset of (b): particle size distribution of the $CoFe_2O_4$ nanoparticles.

Table S1. Comparison of the OER performance of $CoFe_2O_4$@N-CNFs with some previously reported non-precious catalysts in KOH solution.

Number	Electrocatalysts	η (mV)@ 10 mA cm^{-2}	Tafel slop (mV dec^{-1})	Electrolyte	Ref
1	$CoFe_2O_4$@N-CNFs	349	80	0.1 M KOH	This work
2	3D N-doped Graphene/NiCo	>350	614	0.1 M KOH	[S1]
3	$NiCo_2O_4$/G	~400	164	0.1 M KOH	[S2]
4	$Mn_{0.2}Cr_{0.8}O_{1.5}$	~640	~436	0.1 M KOH	[S3]
5	$Co_{0.5}Fe_{0.5}S$@N-MC	410	159	1.0 M KOH	[S4]
6	Co_3O_4 nanotubes	390	76	0.1 M KOH	[S5]
7	$CoFe_2O_4$	~450	N/A	0.1 M KOH	[S6]
8	NiCo-LDH	420	113	0.1 M KOH	[S7]
9	$CoFe_2O_4$/G	460	N/A	0.1 M KOH	[S8]
10	$CoCr_2O_4$/CNS	326	51	1.0 M KOH	[S9]
11	$MnCo_2O_4$@PPY	~480	N/A	0.1 M KOH	[S10]
12	$Co_3O_4/NiCo_2O_4$ DSNCs	~400	110	1.0 M KOH	[S11]

Supporting References:

[S1] S. Chen, J. Duan, M. Jaroniec and S. Z. Qiao, *Angew. Chem. Int. Ed.* **2013**, *52*, 13567-13570.

[S2] D. U. Lee, B. J. Kim, Z. Chen, *J. Mater. Chem. A* **2013**, *1*, 4754-4762.

[S3] X. Song, T. Yang, H. Du, W. Dong, Z. Liang, *J. Electroanal. Chem.* **2016**, *760*, 59.

[S4] M. Shen, C. Ruan, Y. Chen, C. Jiang, K. Ai and L. Lu, *ACS Appl. Mater. Interfaces* **2015**, *7*, 1207-1218.

[S5] H. Wang, S. Zhuo, Y. Liang, X. Han, B. Zhang, *Angew. Chem. Int. Ed.* **2016**, *55*, 9055-9059.

[S6] Y. Xu, W. Bian, J. Wu, J. H. Tian, R. Yang, *Electrochim. Acta* **2015**, *151*, 276-283.

[S7] J. Jiang, A. Zhang, L. Li and L. Ai, *J. Power Sources* **2015**, *278*, 445-451.

[S8] W. Bian, Z. Yang, P. Strasser, R. Yang, *J. Power Sources* **2014**, *250*, 196-203.

[S9] M. Al-Mamun, X. Su, H. Zhang, H. Yin, P. Liu, H. Yang, D. Wang, Z. Tang, Y. Wang, H. Zhao, *Small* ***2016***,*12*, 2866–2871.

[S10] X. Cao, W. Yan, C. Jin, J. Tian, K. Ke, R. Yang, *Electrochim. Acta* **2015**, *180*, 788-794.

[S11] H. Hu, B. Guan, B. Xia, X. Lou, *J. Am. Chem. Soc.* **2015**, *137*, 5590-5595.
